# Modelling the impacts of climate change on faba bean (*Vicia faba* L.) production in Welmera area, central Ethiopia

**DOI:** 10.1016/j.heliyon.2021.e08176

**Published:** 2021-10-14

**Authors:** Girma Asefa Bogale, Mengistu Mengesha Maja, Gebre Hadgu Gebreyohannes

**Affiliations:** a School of Natural Resources Management and Environmental Sciences, Haramaya University, P.O.Box 138, Diredawa, Ethiopia; b Tigray Agricultural Research Institute (TARI), Agronomy/Physiology, Crop Modeling, Mekelle, Ethiopia

**Keywords:** Climate change and variability, Fabab bean, Modelling, GCM, RCP

## Abstract

Climate change is affecting agricultural production and other aspects of life but only limited number of studies took interest in characterizing and projecting climate and its impact on crop production at local level. The threat to agricultural sector is more serious in Ethiopia, where climate is influenced by diverse topography and varying landscape features. This study was conducted in Welmera area to model the impacts of climate change on production of two faba bean (*Vicia faba* L.) varieties (Tums and Gora). Historical climate and crop yield data were obtained from the National Meteorological Agency of Ethiopia and Holeta Agricultural Research Center, respectively. Future climate data were downscaled by an average ensemble of four GCMs (BSS-CSM1-1, HadGEM2-ES, MIROC-ESM, NorESM1-M) in near- and mid-century (2030s and 2050s) under both RCP 4.5 and RCP 8.5. Rainfall by near-century will increase by up to 50% depending on the concentration pathway considered compared to the baseline period (1988–2017). The projected average rainfall total of *belg* season (FMAM) will increase by 88.17% under RCP 4.5 scenario and 95.38% under RCP 8.5 scenario in near-century. The future projection revealed that the highest mean monthly rainfall and temperature changes will occur in July (147.3 mm/month) and August (0.24 °C/month) under RCP8.5. However, in August and September mean monthly rainfall will decrease dramatically by 50.85 % and 31.05% from 2020 to 2079 under RCP 8.5 and RCP 4.5, respectively. The yield of Tumsa variety will decrease by up to 24.19% under RCP8.5 in mid-century. Gora variety will see an increase of yield by 18.24% under RCP 4.5 in mid-century and 28.03% under RCP 8.5 in near-century. Overall, the area will experience an increase and a decrease in faba bean yield for both varieties in the upcoming decades in the study area. Performance evaluation of the models showed that they were able to predict future yield faba bean varieties in the area with acceptable accuracy. Inconsistency of future climate variables and impact on fababean production underscores the need to develop location-specific adaptation strategies. Further studies that consider wider area could be necessary to better understand the impact of future climate on faba bean production in the study area and similar agroecologies in the country.

## Teaser

Climate Change and Variability, Fabab bean, Modelling, GCM and RCP

## 1 Introduction

Climate is one of the main determinants of agricultural productivity mainly in developing countries as smallholders lack capacity to adapt to “hotter and more volatile planet” (Anwar et al., 2013). Agriculture is often regarded as one of the sectors most vulnerable to climate change/variability. The sub-Saharan Africa is the most vulnerable region to climate change as the region already has a large proportion of food insecure population and significant portion of the region's economy relies on rain fed agriculture (Schlenker and Lobell 2010; Connolly-Boutin and Smit, 2016). The unreliable and unpredictable rainfall and warmer temperature adversely influences crop production thereby exacerbating food insecurity of most of the countries in sub Sharan Africa (Abera et al., 2018).

Projected changes in mean temperature and rainfall will exacerbate climate change and associated risks in North-Eastern Africa including Ethiopia (IPCC, 2021). For instance, Muleta (2021) projected an increasing trend for both minimum and maximum temperatures while the same study found no consistent trend in rainfall in Baro-Akobo Basin of Ethiopia in upcoming decades. Conway and Schipper (2011) projected a further warming ranging from 0.7-2.3 °C by 2020s and 1.4–2.9 °C by 2050s in Ethiopia in all four seasons. Similarily, Setegn et al. (2011) found a wide range of regional warming and reduced rainfall in the northern highlands of Ethiopia. Such warming and more frequent and intense drought conditions threaten agricultural production of smallholders that largely rely on rainfed agriculture of subsistence level, and have been a major source of food insecurity in the country (Tesfaye et al., 2017).

In Ethiopia, agriculture is the pillar of the country's economy as it accounts for about half of the country's GDP, employs over 80% of the population, and 90% of foreign currency earnings (World Bank, 2018). However, a range of climatic, edaphic as well as management factors negatively affect productivity and the country's ability to feed its burgeoning population. For example, severe drought of 1983/84 that resulted in famine of immense proportion in the northern Ethiopia (World Bank, 2006) and a recent drought that devastated the livelihoods of farmers of Eastern Ethiopia in 2015 are typical examples of climatic challenges the country faces (Funk et al., 2016; Yuan et al., 2018).

Faba bean (*Vicia faba* L.) is a cool-season crop and grown worldwide as a grain and green-manure legume (Fouad et al., 2013). The crop is used for both human consumption and animal feed as a source of protein and carbohydrate (Salmeron et al., 2010). Moreover, the crop improves soil fertility as it fixes atmospheric nitrogen in large quantities and leaves a lot of N-related yield effect via its large biomass to subsequent crops (Watson et al., 2017). However, climate variability due to rising temperature and erratic rainfall are among the various factors that reduce its productivity in the country. Although faba bean production occupies over 30% of cultivated land of the country, the average national productivity of 1.5 tons/ha (Central Statistical Agency (CSA), 2013) is lower than world average grain yield of 1.86 tons/ha for faba bean (FAOSTAT, 2018). Such low productivity in faba bean yield could be attributed to a range of factors including climate variability/change, soil acidity, water logging, enhanced disease and pest development (Khan et al., 2010; Bishop et al., 2016; Terefe et al., 2016).

Welmera district is one of the areas that have been suffering as a result of climate change and variability. Multiple biophysical factors including climatic, edaphic and management factors interact to adversely affect crop production in the area. A recent study pointed out that rainfall is the most important determinant factor of production levels of various crops in central Ethiopia (Alemayehu and Bewket, 2017). Changes in rainfall and temperature are production challenges that affect phenological maturity and grain yield of faba bean production, which is one of the staple crops in the area. Previous studies established relationship between crop production and historical climate data in Ethiopia and found a mixed result (Abera et al., 2018). Despite the impacts of current and future climate change on faba bean production, no studies projected future climate and assessed its impact on faba bean production at local level. Therefore, the aim of this study was to assess the likely changes of future rainfall and temperature and evaluate how these changes will impact faba bean production in Welmera area, central Ethiopia.

## 2 Materials and methods

### 2.1 Description of the study area

**Figure 1:**
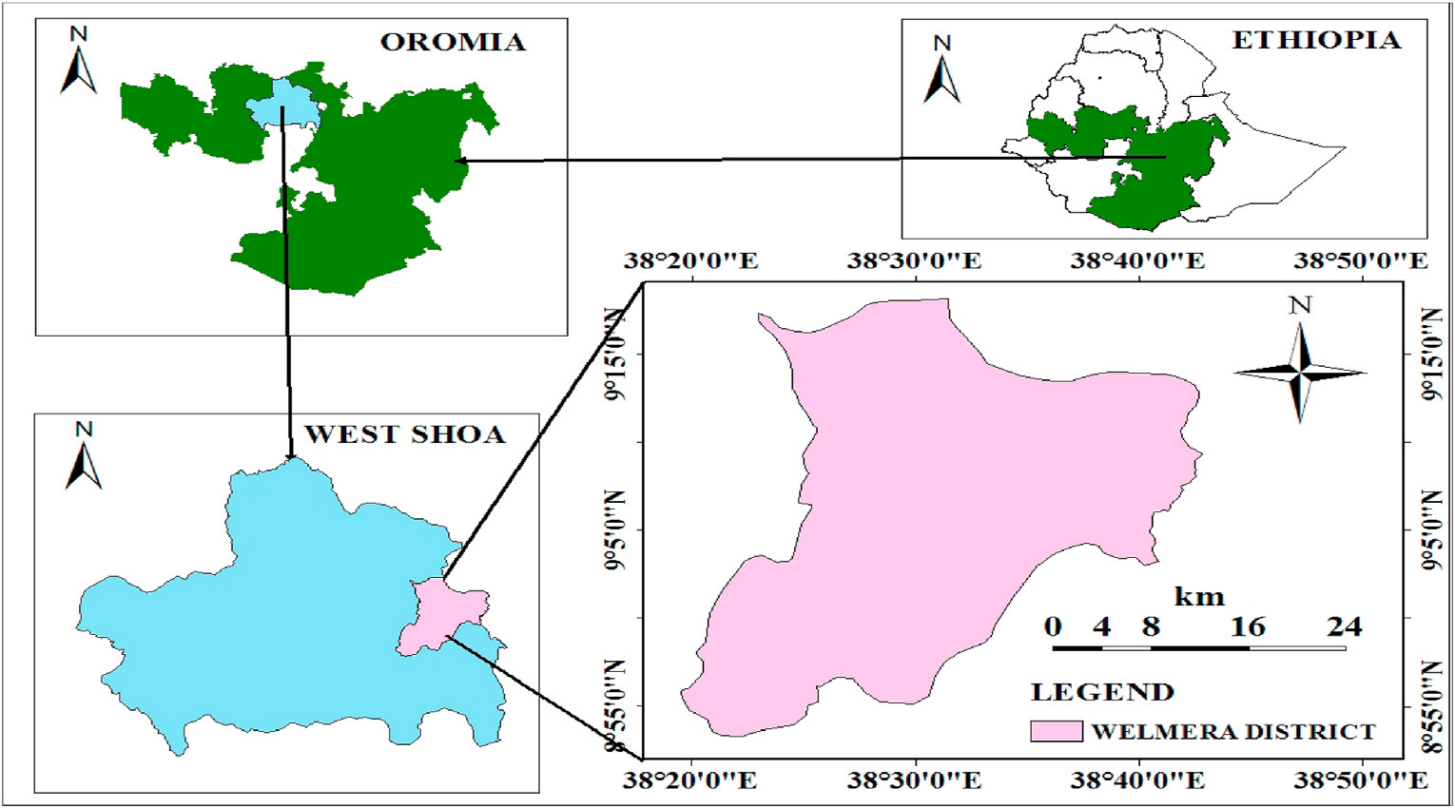
Map of the study area.

The study was conducted in Welmera area, which is located in Oromia Regional State, Central Ethiopia (Figure 1). The district is located 30 km west of Addis Ababa, the capital city of the country along Ambo road. The area has an altitude ranging from 2000-3380 m.a.s.l. (Bureau of Agriculture, 2013). Geographically, the district is located at 09^o^ 02′ 34″N to 09^o^ 06′ 46″N and 43^o^02′ 02″E to 43^o^05′ 38″E. The area is bordered with Sululta district in the north, Sebeta-Awas district in the south, Burayu City Administration in the west and Ejere district in the east.

**Figure 2:**
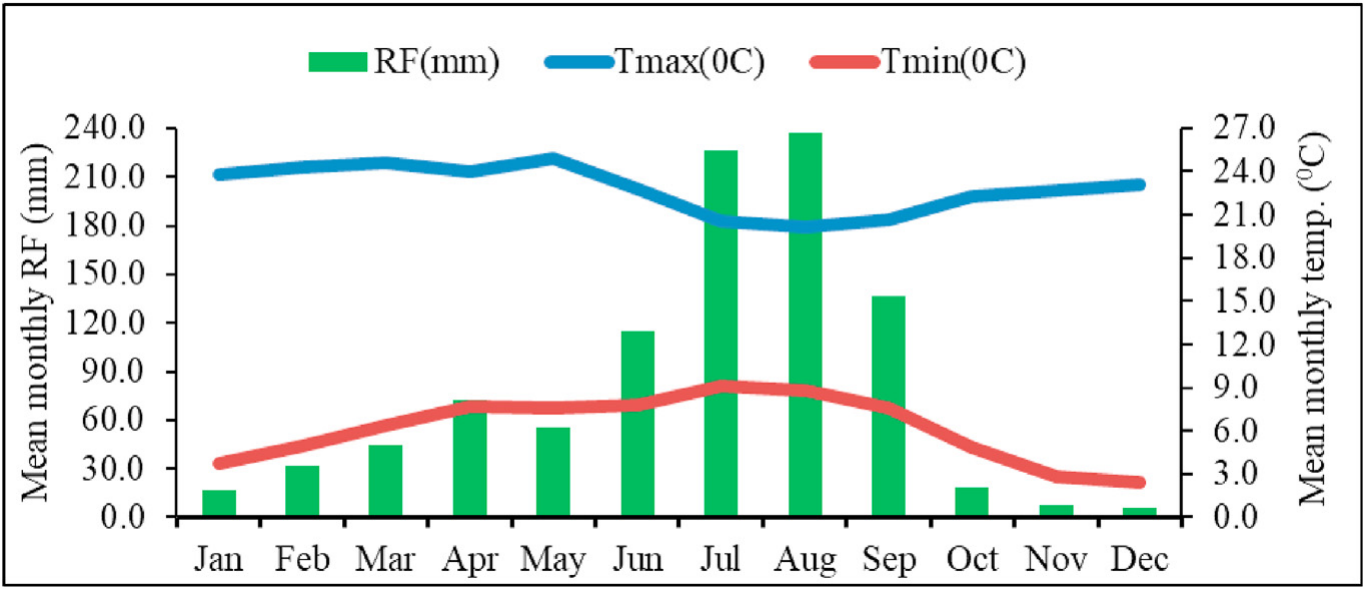
General description of climate of Welmera area (1988–2017).

The area has two agro-ecological zones, namely *dega* (61%) and *woynadega* (39%) (Bureau of Agriculture, 2013). The area is characterized by bimodal rainfall pattern (Figure 2) with mean annual rainfall of 970 mm. The main rainy season (*kiremt*) spans from June to September and accounts for 70% of the rainfall, while the remaining 30% is received during short rainy season (*belg*), which spans from February to April (Bureau of Agriculture, 2013). The mean maximum, minimum and mean annual temperature of the study area were 22.8, 6.2, and 14.5 °C, respectively.

Agriculture is the main livelihood of the study area. The major farming system in the area is mixed crop-livestock production where crop production and livestock rearing take place simultaneously. The major crops grown in the area include *teff*, wheat, barley and faba bean, while the livestock rearing include cattle, sheep, goats, equines and poultry production. The majority of the land of the district (65.83%) is under cultivation. Natural vegetation covers about 19.64% of the land area of the district, while the remaining 4.49% and 10.01% are grassland and other land uses, respectively (Table 1).

**Table 1:** The general land use and land cover of Welmera area.

No.	Description	Area (km^2^)	%
1	Land under crop	339	65.83
2	Land covered by vegetation	101	19.64
3 4	Grassland Assigned for other purposes	23 52	4.49 10.01
Total		515	100

Source: Office of Agricultural Development of Welmera, 2010

### 2.2 Data collection

#### 2.2.1 Historical climate data

In this study, historical climate data for the period of 1988–2017, which were considered as baseline climate data for Welmera area were obtained from National Meteorology Agency of Ethiopia. The climate data include rainfall (mm), maximum and minimum temperature (^0^C) and solar radiation (MJ/m^2^/d).

#### 2.2.2 Downscaling future climate data

Site specific future climate data were downscaled from an average ensemble of four GCMs (BSS-CSM1-1, HadGEM2-ESM, MIROC-ESM, NorESM1-M) under RCP 4.5 (medium) and RCP 8.5 (high) concentration pathways in time period of centered 2030s (2020–2049) and 2050s (2050–2079) using MarkSim weather generator (Jones et al., 2003; Jones and Thornton, 2013). MarkSim is currently used to downscale output from GCMs and generate daily rainfall, solar radiation, minimum and maximum temperature at a specific site (Jones and Thornton, 2000). It requires geographical coordinate and altitude to downscale and generate daily future data of a given site (Jones and Thornton, 2013). The model is linked with Google Earth to indicate the target location and has options to use different GCMs. In this study, the models were selected based on previous information that they are highly applicable for African climate studies (Thornton, 2003; Yang et al., 2014) and have been statistically bias corrected (Dosio and Paruolo, 2011).

### 2.3 Soil data

Soil data of the study area were acquired from HARC (2015). The major soil type of the study area is Nitisol, which is red soil. Physical and chemical properties were analyzed using soil samples collected from a pit of 180cm deep following appropriate laboratory procedures. The analysis includes the drainage characteristics and physico-chemical parameters such as texture, bulk density, soil pH, saturated soil water content, lower limit (LL), drained upper limit (DUL), initial soil water content, relative root distribution, total nitrogen (TN), organic carbon and organic matter (Ritchie et al., 1998). The soil characteristics at each depth of the study area are shown in Table 2.

**Table 2:** Physical and chemical soil properties of the Welmera area.

Parameters	Depth (cm)			
	0–20	20–40	40–80	80–180
Texture (%)	c-	c-	c-	c-
Sand	34.1	30.2	27.5	28.8
Silt	28.0	24.0	22.0	22.0
Clay	66.0	74.0	76.0	72.0
Bulk density (g/cm³)	1.35	1.35	1.33	1.34
Organic carbon (%) pH	5.4	6.0	6.0	5.9
DUL	0.463	0.463	0.47	0.467
DLL	0.396	0.445	0.456	0.432
SWC RGF	0.532 1	0.57 0.549	0.579 0.301	0.559 0.165
TN	0.17	0.13	0.11	0.08

TN; Total Nitrogen (%), C; Clay UDL/LDL; Upper Drainage Limit and Lower Drainage Limit (meter) SWC; Saturated soil water content (kg water/kg dry soil), RGF; Root growth factors (Rhizometrics) (0.0 up to 1).

### 2.4 Description of experimental materials

Eight years crop data were obtained from HARC for two improved faba bean varieties, Gora and Tumsa. The varieties were selected based on the availability of long term data and their difference in maturity period. According to the report obtained from HARC, Tumsa and Gora varieties were released in 2010 and 2013, respectively in the study area. The spacing within and between rows were 10 cm and 40 cm, respectively. Besides this, 100 kg/ha of diammonium phosphate (DAP) was applied at the time of sowing.

## 3 The DSSAT model evaluation

In this study, the Decision Support System for Agro technology Transfer (DSSAT), which is a software package that incorporates independent models for more than 25 different crops with a program that facilitates the evaluation and application of crop models for different purpose was employed (Hoogenboom et al., 2012b: Jones et al., 2003). DSSAT is a suit of crop models developed to simulate growth, development and yields of several crops and changes in soil water, carbon and nitrogen that take place under the cropping system over time (Jones et al., 2003). Therefore, DSSAT crop model was selected due to the fact that it has been successfully used worldwide in a broad range of conditions and purpose (Harb et al., 2016). There are lots of different crops simulated with cropping simulation model (CSM), including maize, wheat, rice, barley, sorghum, maize, peanut, dry bean, chickpea, cowpea, faba bean, potato, tomato, and, cabbage etc. In this study, the CROPGRO-Faba bean model (Boote et al., 2002), which is embedded within DSSATv4.7 (Hoogenboom et al., 2009) was used to simulate daily phenological development, growth and yield of faba bean in response to environmental and management factors.

### 3.1 Model calibration

Model calibration is the adjustment of parameters so that simulated values compare fairly well with observed ones (Timsina and Humphreys, 2006). In the CROPGRO-Faba bean model, species, eco-type parameters and genetic cultivar coefficients were required for defining the traits that differentiate between cultivar within crop species (Jones et al., 2003). DSSAT crop model was calibrated and evaluated using experimental data (2014 up to 2017) obtained from HARC. Cultivar specific crop parameters such as days to flowering, days to physiological maturity and grain yields of both varieties were fixed. The CROPGRO-Faba bean model in DSSAT requires a set of eighteen (18) genetic cultivars of specific parameter for its calibration (Table 3). As the result, ten of them (PPSEN, EM-FL, F1-SH, F1-SD, SD-PM, F1-LF, LEMAX, PODUR, SDPRO and SDLIP) were controlled the timing of phenological stages, and the remaining eight (CSDL, SLAVR, SIZLF, XFRT, WTPSD, SFDUR, SDPDV, THRSH) characterize the potential yield under optimal condition.

**Table 3:** Genetic coefficients and definition used to calibrate and validate the CROPGRO-Faba bean model for determination of Gora and Tumsa varieties in HARC.

Symbols	Definitions	Gora	Tumsa
CSDL	Critical short day length which reproductive development progress with no day length effect (for short day plants) (hour)	8.45	7.35
PPSEN	Slope of the relative response of development to photoperiod with time (positive for short day plants) (1/hour).	-0.08	-0.06
EM-FL	Time between plant emergence and flower appearance (R1) (photothermal days)	15.8	16.7
Fl-SH	Time between first flower and first pod (R3) photothermal days)	11.5	11.5
F1-SD	Time between first flower and first seed (R5) (photothermal days)	35.1	36.6
SD-PM	Time between first seed (R5) and physiological maturity (R7) (photothermal days)	38.80	35.5
F1-LF	Time between first flower (R1) and end of leaf expansion (photothermal days)	75.55	78.65
LFMAX	Maximum leaf photosynthesis rate 30 °C, 350vpm CO_2,_ and high light (mg/CO^2^/m^2^-S)	1.00	1.00
SLAVR	Specific leaf area of cultivar under standard growth conditions (cm^2^/g	375	385
SIZLF	Maximum size of full leaf (three leaflets) cm^2^	103.8	104.7
XFRT	Maximum fraction of daily growth that is partitioned to seed + shell	7.95	6.85
WTPSD	Maximum weight per seed (g)	15.75	15.25
			
SFDUR	Average seed per pod under standard growing conditions ((#/pod)Time required for cultivar to reach final pod load under optimal conditions (photothermal days)	28.0	27.5
SDPDV	Average seed per pod under standard growing conditions ((#/pod)	8.4	8.4
PODUR	Time required for cultivar to reach final pod load under optimal conditions (phototermal days)	5.0	5.0
			
THRSH	The maximum ​ratio of (seed/ (seed+shell)) maturity Causes seed to stop growing as their dry weights increases until shells are filled in a cohort	85.8	84.5
SDPRO	Fraction protein in seeds (g (protein)/g (seed))	0.42	0.42
SDLIP	Fraction of oil in seeds (g (oil)/g (seed))	0.02	0.02

The genetic coefficients of each variety (Table 3) were determined manually by running the model iteratively (Godwin et al., 1989). The model was run first to fix the genetic coefficients (cultivar parameters) that influence anthesis. Then, the cultivar parameters that control maturity and yield components were set in that order.

Model calibration was conducted by comparing the simulated values of development and growth characteristics of each crop with their corresponding observed values and by calculating statistical parameters like R^2^, RMSE, RMSEn and d-stat that evaluate the agreement between observed and simulated values (Andarzian et al., 2011).

### 3.2 Model validation

The performance of the model for both crops was validated using an independent crop data obtained from HARC during the rainy period (2010–2013). In order to evaluate the performance of the model, statistical parameters such as coefficients of determination (R^2^), root mean standard error (RMSE), and normalized root mean standard error (RMSEn) were used. The index of agreement (d-stat) was also computed to measure the goodness of fit between measured and simulated values (Wilmot *et al.*, 1985). The comparison has been done with simulated mean values of days to anthesis, days to maturity, and grain yield (kg/ha) measured values. RMSE quantifies the patterns of similarity between values predicted by the model and values actually observed were calculated as explained by Loague and Green (1991) with the help of the following equation;

(Equation 1) $$\text{RMSE}=\sqrt{\frac{\sum_{\text{i}=1}^{\text{N}}{\left(\text{pi}-\text{oi}\right)}^{2}}{\text{n}}}$$

where, RMSE is root mean square error, n is the number of observations, Pi and Oi are predicted and observed values for i^th^, measurement. Thus, lower values indicate good fit of the model. M is the overall mean of observed value.

In addition to this, normalized root mean square error (RMSEn**)** was also computed as;

(Equation 2) $$\text{RMSEn}=\sqrt{\frac{\sum_{\text{i}=1}^{\text{N}}{\left(\text{pi}-\text{oi}\right)}^{2}}{\text{n}}}\text{ ​x ​}\frac{100}{\text{M}}$$

where, RMSEn is normalized root mean square error and M is the overall mean of observed values. RMSEn (%) gives a measure of the relative difference of simulated versus observed data. The simulation is considered excellent if the RMSEn is less than 10%, good if it is greater than 10% and less than 20%, fair if RMSEn is greater than 20% and less than 30%, and poor if the RMSEn is greater than 30% (Valizadeh et al., 2013).

The index of agreement (d-statistic) and RMSEn were used for time series data because RMSEn is normalized and reduces variability with time, although the d-statistic provides a single index of model performance that encompasses bias and variability, and is a better indication of 1:1 prediction than R^2^ (Anothai et al., 2008). According to the d-statistic, the closer index values to unity, the better the agreement between the two variables that are being compared and vice versa (Anothai et al., 2008; Andarzian *et al.*, 2011). The d-statistics was computed as;

(Equation 3) $$\text{d}=1-\left[\frac{\sum_{\text{i}=1}^{\text{n}}{\left(\text{pi}-\text{oi}\right)}^{2}}{\sum_{\text{i}=1}^{\text{n}}{\left(\left|{\text{p}}^{\text{′}}\text{i}\right|+\left|{\text{o}}^{\text{′}}\text{i}\right|\right)}^{2}}\right]$$

where, n is the number of observations, Pi the predicted value for i^th^ measurement, Oi is a observed values for i^th^ measurement, Pʹi = Pi −M and Oʹi = Oi−M (M is the overall mean of observed variable). So if the d-statistic value is closer to one, then there is good agreement between the two variables that are being compared and vice versa. However, linear regression was applied between simulations and observations to evaluate model performance coefficient determination (R^2^) for each simulation (Loague and Green, 1991).

(Equation 4) $${\text{R}}^{2}=\frac{\text{SS}\left(\text{regression}\right)}{\text{SS}\left(\text{residual}\right)}\;$$

where, SS (regression) is sum square regression and SS (residual) is sum square of residual.

### 3.3 Simulation of faba bean yield

The integration of different treatments in DSSAT model offers options that enable efficient analysis of selected cultivars under different scenarios (RCP 4.5 and RCP 8.5) and time slices (2030s and 2050s). The simulation of faba bean yield change under future climate change impact assessments was represented in 2030s and 2050s. For comparison purposes, the model simulated yield of both varieties for the last three decades (1988–2017), and mean yield was used to represent the baseline data. In this study, daily weather data of historical and downscaled future scenarios were employed to produce baseline yield change and future climate projection. Therefore, yield gap analysis encompasses quantifying the differences between simulated potential yield and baseline levels and identifying those factors responsible for the yield differences (Liang et al., 2011; Belay, 2014). Baseline yields were used as the references for calculating yield gaps in the future time slices. Finally, the yield percentage change was compared with the baseline as calculated using the following formula;

(Equation 5) $$\text{Δ}\text{Yield}=\text{ ​}\frac{\text{Ysimulated}-\text{Yobserved}}{\text{Yobserved}}\text{ ​x}100$$

where Ysimulated is simulated yield (kg ha^−1^), Yobserved is yield of the observed period (kg ha^−1^) and Δyield is the yield difference (%).

## 4 Result and discussion

### 4.1 Climate projection

#### 4.1.1 Projection of annual rainfall and temperature

Table 4 shows projected future climate under RCP 4.5 and (RCP 8.5 concentration pathways in near-century (2030s) and mid-century (2050s) compared to baseline period of 1988–2017. The annual rainfall, maximum and minimum temperature will increase substantially in both scenarios in the study area. Accordingly, annual rainfall is expected to increase by 36.27% and 51.57% at Welmera area in near-century under RCP 4.5 and RCP 8.5 scenarios, respectively. Similarly, annual rainfall will increase by 44.67% and 48.36% in mid-century under RCP 4.5 and RCP 8.5 scenarios, respectively. Various studies projected a higher rainfall in central highlands of Ethiopia. For instance, Shongwe et al. (2011) forecasted a wetter East African region while Vizy and Cook (2012) projected an increase in heavy precipitation and occurrence of extremely wet days in the region by 2050s. Similarily, rainfall is expected to increase by 12–69% in the central rift valey during kiremt season (Muluneh et al., 2015), while similar projection of heavy precipitation was reported for Eastern Africa by the Intergovernmental Panel on Climate Change (IPCC, 2021). These findings suggest that low annual rainfall might not be the main threat of faba bean production in the area. However, extreme rainfall events in certain growing months could lead to water-logging, even if only transient, which could adversely affect faba bean production (Gemechu et al., 2001). Furthermore, excessive rainfall during faba bean growing season may increase annual run off (up to 40% according to IPCC) thereby leading to loss of fertile soil and reducing crop yield.

**Table 4:** Projected (%) changes in annual rainfall (mm) and temperature (^0^C) from near- and mid-century under RCP 4.5 and RCP 8.5 scenarios compared to baseline (1988–2017).

Scenarios	Rainfall (%)	Max.Temp (^0^C)	Min.Temp (^0^C)
Baseline	915 mm	22.8	6.2
RCP4.5 near-century RCP4.5 mid-century RCP8.5 near-century RCP8.5 mid-century	+36.27% +44.67% +51.57% +48.36%	+0.12 +0.10 +0.08 +0.14	+0.30 +0.47 +0.40 +0.54

On the other hand, Tadese *et al.* (2020) reported a decline in mean precipitation during *kiremt* season that contributes the majority of annual rainfall in Awash river basin under RCP 8.5 by 2050s. Using multiple GCMs, Setegn et al. (2011) reported inconsistent trend of future rainfall total at Adet station in Amhara Regional State. This implies that uncertainties remain and the direction and magnitude of rainfall projection depend on the types of models employed to generate the future data (Conway and Schipper, 2011). The spatial variation of both historical and future annual rainfall stems from the fact that Ethiopia has diverse topography and experiences seasonal migration of intertropical convergence zone and associated atmospheric circulations (Fazzini et al., 2015). Moreover, alteration of monsoons and the El Nino-Southern Oscillation of the Pacific Ocean are important drivers of climate variability in the region (Conway, 2009).

#### 4.1.2 Seasonal rainfall total

Projected seasonal rainfall total will increase in both 2030s amd 2050s under both scenarios (Table 5). *Kiremt* (JJAS) seasonal rainfall total in near-century will increase by 22.65% and 38.31% under RCP 4.5 and 8.5 scenarios, respectively. Similarly, by mid-century, *kiremt* rainfall will increase by 32.37% under RCP 4.5 and 40.23% under RCP 8.5. *Kiremt* season is the main growing season; wetter than usual condition during this season will ensure adequate soil moisture suitable for faba bean production in the upcoming decades in the study area. However, excessive rainfall during this season may cause waterlogging and constrain faba bean production despite the plant's ability to tolerate waterlogged condition through improved root porosity (Solaiman et al., 2007). During near-century, a substantial increase of 88.17% and 95.38% in rainfall total is projected for *belg* (FMAM) season under RCP4.5 and RCP8.5, respectively. A similar increase of 91.49% is projected during *belg* season in mid-century under RCP 4.5. *Belg* rainfall total could reach up to 388.79 mm under RCP 4.5 and then decline to 381.97 mm under RCP 8.5. This finding shows that rainfall is expected to increase more during *belg* season than *kiremt* season in the study area in the coming decades. Furthermore, *bega* rainfall totals will be higher under RCP 8.5 than RCP 4.5 scenarios than the baseline period.

**Table 5:** Projected changes in average seasonal rainfall total (%) in near- and mid-century under RCP 4.5 and RCP 8.5 scenarios compared to baseline (1988–2017).

	*Kiremt* (JJAS)	*Belg* (FMAM)	*Bega* (ONDJ)
Baseline	715	205	50
RCP4.5 near-century RCP4.5 mid-century RCP8.5 near-century RCP8.5 mid-century	+22.65% +32.37% +38.31% +40.23%	+88.17% +91.49% +95.38% +77.63%	+18.08% +28.37% +61.54% +44.57%

**Figure 3:**
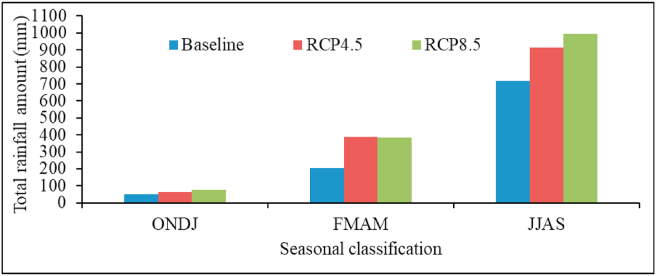
Comparison of projected average seasonal rainfall with the baseline period under RCP 4.5 and RCP 8.5 scenarios.

In ​the ​sameway, ​*bega* ​seasonal ​rainfall ​will ​significantly increase by near- and mid-century under both scenarios. The rate of increase in *bega* rainfall total will be 18.08% and 61.54% in 2030s under RCP 4.5 and 8.5 scenarios. Similarily, in mid-century, *bega* rainfall will increase by 28.37% and 44.57 ​% under RCP 4.5 and 8.5, respectively (Figure 3). The timing of seasonal cycle determines the length of growing season and agricultural yields (Vizy et al., 2015).

#### 4.1.3 Monthly rainfall

**Figure 4:**
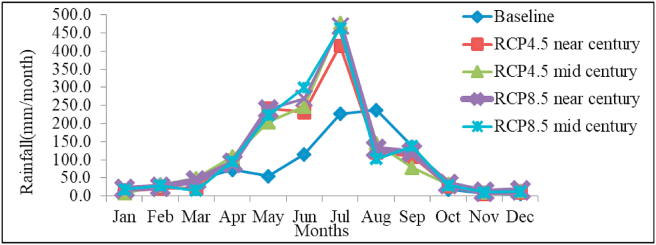
Comparison of mean monthly baseline rainfall with model predicted under RCP 4.5 and RCP 8.5 scenarios.

The mean monthly rainfall by 2030s and 2050s compared to the baseline period under RCP 4.5 and 8.5 scenarios is shown in Figure 4. The result showed the highest increase in monthly rainfall under both scenarios and time slices will occur during the months of May and July as compared to the baseline. August will have a slightly lower rainfall than the baseline in near- and mid-century under both RCPs. Cheung et al. (2008) and Negash et al. (2013) also reported a statistically significant rainfall decline during some months of *kiremt* season at watershed level in different parts of Ethiopia including the central highlands. Although the mean rainfall total of *kiremt* is projected to increase, the projected decline in August may have adverse impact on faba bean production as it may coincide with critical growth/developmental stages.

#### 4.1.4 Rainfall projection during faba bean growing season

**Figure 5:**
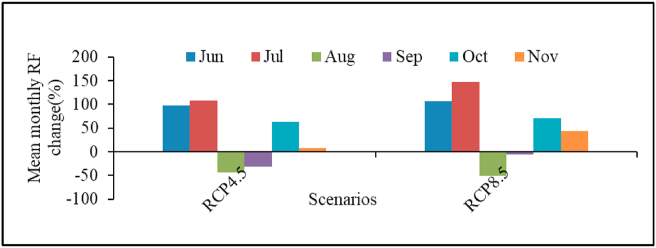
Model predicted changes in mean monthly rainfall distribution during faba bean growing season under RCP 4.5 and 8.5 scenarios.

In the study area, faba bean grows from June to November (Table 6). The mean rainfall in the months of June and July is projected to increase dramatically in near- and mid-century under both scenarios (Figure 5). These months are very critical for faba bean growth, as they encompass major growth stages. The highest rainfall during faba bean growing season will be in July under RCP 4.5 and RCP 8.5, whereas the mean monthly rainfall of August will decrease drastically in near- and mid-century under both scenarios. This implies that there will be temporal shifts of monthly rainfall amounts from the baseline period under the two scenarios, which could affect plant performance.

**Table 6:** Projected average of monthly rainfall totals deviation in (%) from the baseline during faba bean growing season in 2030s and 2050s under RCP 4.5 and RCP 8.5 scenarios.

	June	July	August	September	October	November
Baseline	115.1	226.4	237.3	136.5	18.6	7.9
RCP4.5 near-century RCP4.5 mid-century RCP8.5 near-century RCP8.5 mid-century	+83.8 +110.4 +107.1 +104.9	101.0 114.4 134.4 160.2	-49.2 -38.9 -44.9 -56.8	-19.8 -42.3 -11.9 +0.5	+50.5 +76.1 +79.9 +61.6	-13.8 +27.6 +51.6 +35.7

A decrease in seasonal rainfall amount in 2050s during the growing season particularly during the critical reproductive stage is reported over the highlands of Ethiopia (Cairns et al., 2013). Such decline and fluctuation may have negative impact on physiology of faba bean plant and its yield. Infact, Karamanos and Gimenez (1991) found a decrease in leaf area, net photosynthesis, light use efficiency, pod retention, grain filling and distorting of hormonal balance in faba bean due to climate variability in the form of water stress. In general, rain fed faba bean production in 21^st^ century under the changing climate may require possible adjustment of planting time and other management practices to maintain or improve production.

Besides climate variables, several factors contribute to low productivity of faba bean, including soil acidity and fertility decline, frequent disease occurrence, parasitic weeds and lack of high yielding varieties (Getachow, 2006). Fekadu et al. (2018) noted adverse effect of soil acidity and associated nutrient availability on faba bean production in Ethiopian highlands.

### 4.2 Projection of temperature in the study area

#### 4.2.1 Mean annual maximum and minimum temperature

The annual mean maximum is expected to ge warmer in the study area under both RCPs than the baseline period with magnitude of change varying depending on the emission scenario considered. The annual mean maximum temperature of the study area will increase by 0.12 °C and 0.10 °C in near- and mid-century under RCP 4.5 scenario. James and Washington (2013) also showed an increase in temperature during twenty first century in Africa. A similar study found an annual mean maximum temperature increase of just under 1 °C in near-term up to 2 °C in end of the century under RCP4.5 scenario in central Ethiopia (Abera et al., 2018). Under both emission scenarios, higher increase in annual mean minimum temperature is expected in mid-century than near-century. An increase of 0.47 °C and 0.54 °C is projected to occur under both RCP4.5 and 8.5 scenarios in mid century (Table 4). Given that faba bean is a cool season crop, high temperature could affect reproductive development, shorten the growth stage of the crop and reduce legume yield due to low assimilation of photosynthetic products (Kaushal et al., 2013).

#### 4.2.2 Mean monthly maximum and minimum temperature

**Figure 6:**
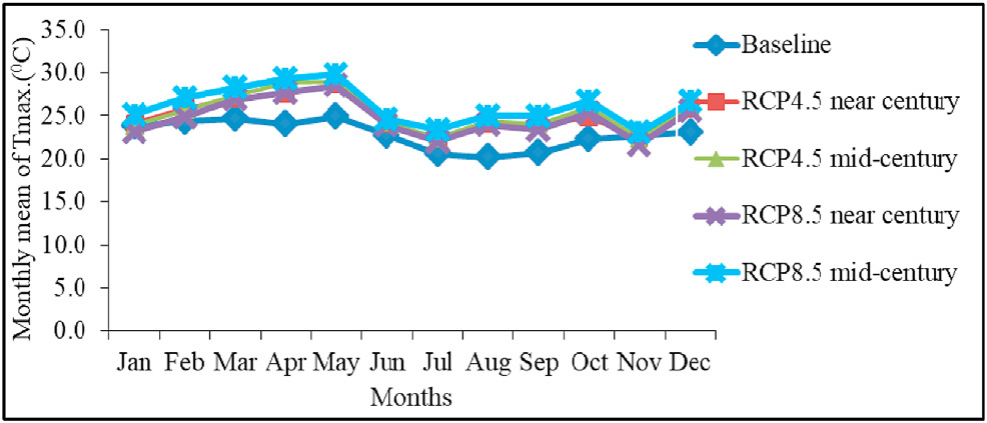
Model predicted and baseline monthly mean of maximum temperature in near and mid-century under RCP 4.5 and RCP 8.5 scenarios.

The monthly mean maximum temperature is expected to increase substantially during both time slices and emission scenarios compared to the baseline (Figure 6). The highest mean maximum temperature increment will be in mid-century under RCP 8.5 scenario. In most cases, mean maximum temperature will be higher than baseline temperature suggesting that the area is likely to warming in the coming decades. Poor yield could be recorded due to late sowing and high temperature as noted by Manning (2017).

**Figure 7:**
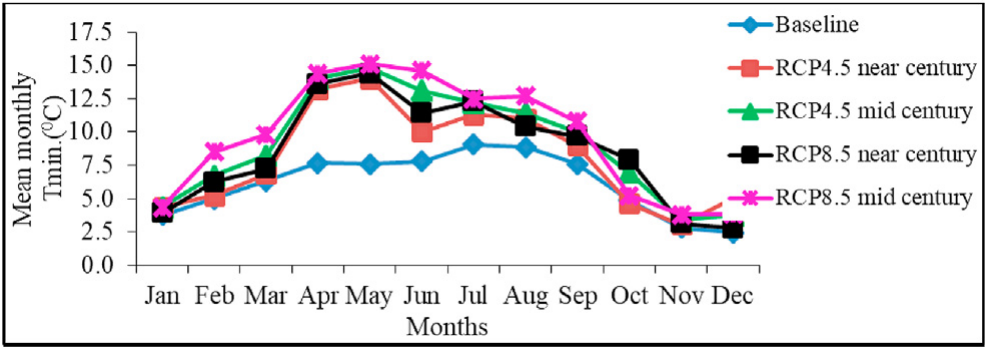
Model projected and baseline mean monthly minimum temperature in near and mid-century under RCP 4.5 and RCP 8.5 scenarios.

Mean monthly minimum temperature will rise to 15.1 °C in May, which is higher than the mean minimum temperature of 9.1 °C recorded in July for the baseline period (Figure 7). This implies that minimum temperature is expected to rise during the months of *belg* and *kiremt* seasons. Similarly, Kassie *et al.* (2014) reported warmer temperature in the near future in central rift valley of Ethiopia. The more pronounced increment of minimum temperature relative to maximum temperature also contributes more to the increase in annual mean temperature. The result is in agreement with the findings of Conway et al. (2004) and Daniel et al. (2014), who reported increasing minimum temperature in various parts of Ethiopia.

#### 4.2.3 Temperature projection during faba bean growing season

**Figure 8:**
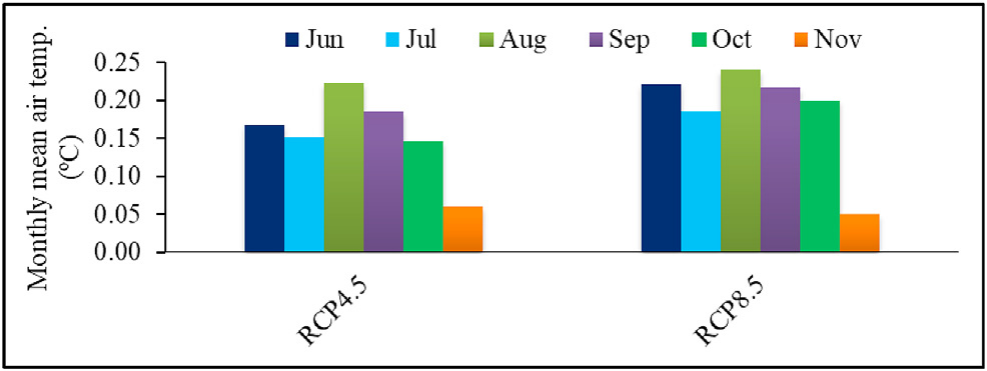
Projected changes in mean monthly air temperature during faba bean growing period under RCP 4.5 and 8.5 scenarios.

The study area is expected to experience higher mean temperature in both time slices and emission scenarios (Figure 8). August will be the warmest month of faba bean growing season, with overall rise in temperature being slightly higher under RCP 8.5 than RCP 4.5. A rise in temperature could lead to increase in evapotranspiration (Zeray et al., 2007; Conway and Schipper, 2011). High temperature also creates more suitable condition for disease and pest development, which could affect faba bean production (Khan et al., 2010). Temperatures events higher than normal are expected to shorten the grain filling period, reduce pollen viability, grain weight and crop yields (Boote and Sinclair, 2006; Hatfield et al., 2011).

### 4.3 Model calibration and performance evaluation for Gora and Tumsa varieties

The performance of CROPGRO model during calibration period is depicted in Table 7. The result revealed that normalized root mean square error of Gora varieties were 7.70% days for flowering, 4.02% days for maturity, and 17.55% for grain yield. Based on the scale of Valizadeh et al. (2013), the model showed excellent performance in simulating days to flowering, days to physiological maturity while, the grain yield had a good agreement between simulated and observed values.

**Table 7:** Comparison of mean simulated and observed days to anthesis, days to maturity and grain yield of faba bean varieties during model calibration.

Variable name	Faba bean varieties											
	Gora						Tumsa					
	Ob	Sim	R^2^	RMSE	RMSEn	d-stat	Ob	Sim	R^2^	RMSE	RMSEn	d-stat
DF	48	50	0.52	3.78	7.70	0.816	50	49	0.70	3.16	6.38	0.906
DM	142	142	0.84	5.70	4.02	0.742	126	125	0.68	1.32	1.05	0.848
Yield (kg/ha)	2380	2212	0.62	403.01	17.55	0.946	2458	2440	0.87	278.67	11.38	0.960

Note: DF; Days to flowering, DM; Days to maturity period.

Similarly, days to flowering and maturity had excellent agreement between simulated and observed values for Tumsa variety but, grain yield showed good agreement. The overall, d-statistics for both varieties showed strong agreements between simulated and observed days to anthesis, days to maturity and yields (kg/ha) over the study area (Table 7). Wilmott *et al.* (1985) reported that the closer d-statistic to 1, the better the simulations.

**Figure 9:**
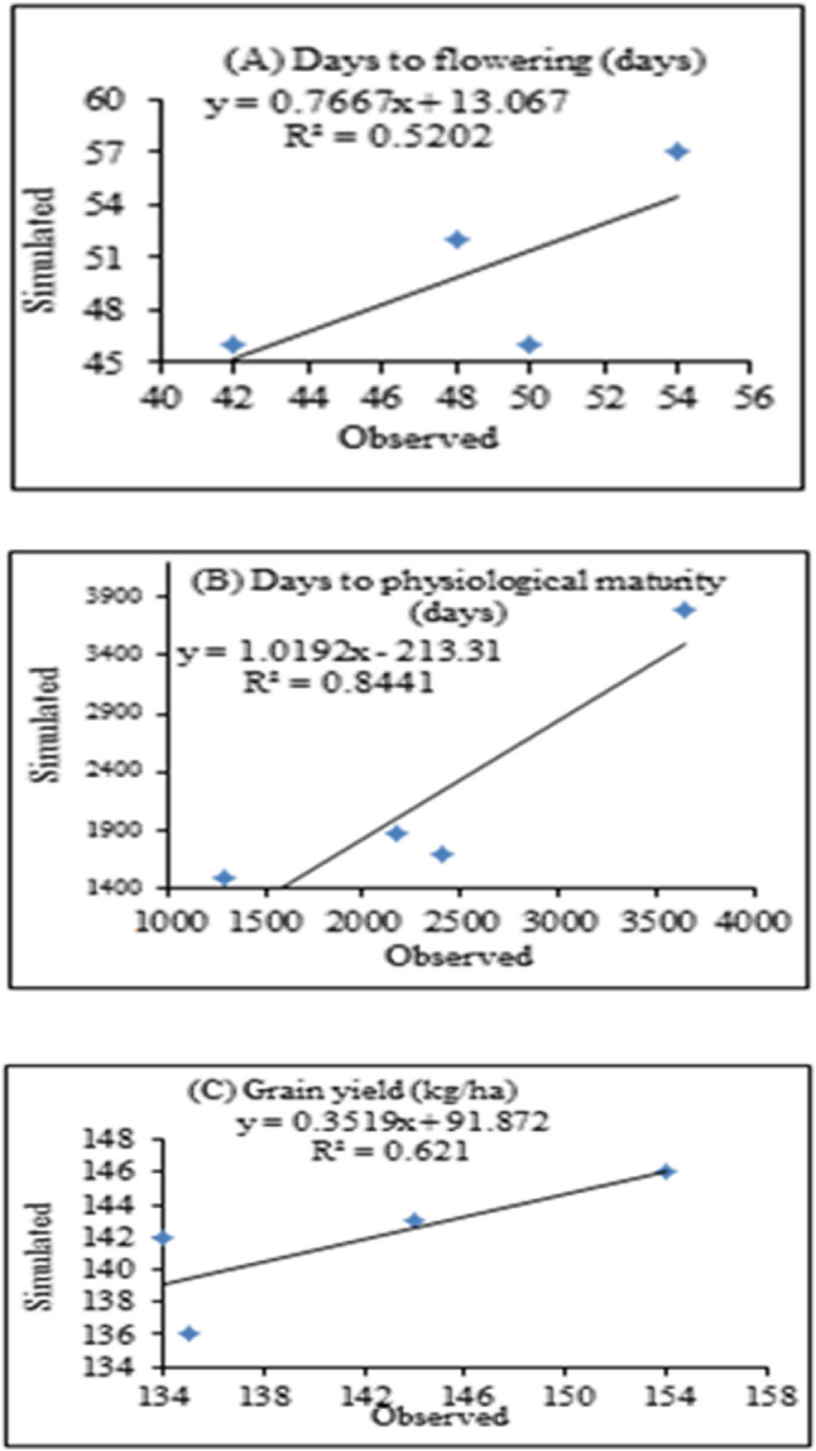
Observed and simulated calibrations for days to flowering, days to physiological maturity and grain yield for Gora variety.

The regression model has a high coefficient of determination (R^2^) indicating that the model performed well under the test of environment (Figure 9). The R^2^ values for Gora variety were 52%, 84% and 62%, for days to flowering, maturity and yield (kg/ha), respectively.

**Figure 10:**
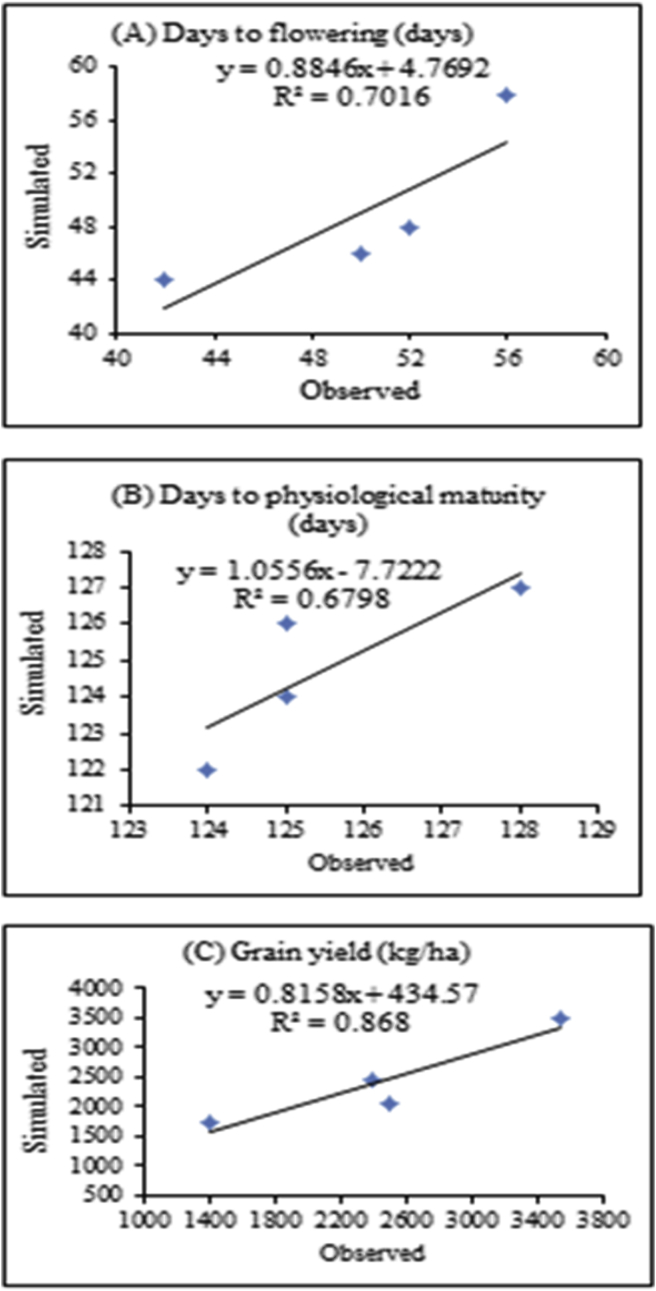
Observed and simulated calibration for days to flowering, days to physiological maturity and grain yield for Tumsa variety.

Figure 10 revealed the performance of DSSAT model in terms of observed versus simulated parameters of Tumsa variety. Tumsa variety also had high the goodness of fit (R^2^) value, which implies that the regression accounts for majority of the variation. It also indicates that there was a strong agreement between observed and simulated data. The coefficient of determination for days to anthesis, days to maturity and grain yield were 70%, 68%, and 87%, respectively. This indicates the model was able to simulate the yield and phenology of the cultivars adequately.

The performance of model after calibration was satisfactory and the results were similar to that of Thornton (2003) and Ma et al. (2006). The model, in the present study found non-limiting conditions of water and nitrogen, which are the major environmental limitations of faba bean in Welmera area and the surrounding central highlands of Ethiopia.

### 4.4 Model validation for CROPGRO-Faba bean varieties (Gora and Tumsa)

The simulated mean for days to physiological maturity 125 days (Gora) and 133 days (Tumsa) are highly comparable to the observed values of 126 and 232, respectively (Table 8). The two varieties have differences in days to maturity, which is in line with the finding of Wondimu (2016), who observed a variation for days to maturity between faba bean varieties. The root mean square error percentages for days to flowering, days to physiological maturity and grain yield for Gora variety were 2.95%, 1.54% and 8.68%, respectively (Table 8). A similar trend was found for Tumsa variety with error percentages for days to flowering, physiological maturity, and grain yield of 2.92%, 0.99% and 8.98%, respectively. Overall, CROPGRO-model had an excellent performance in simulating days to flowering, days to physiological maturity and grain yield. Moreover, the d-statistic was in good agreement between simulated and observed values of both varieties of faba bean for grain yield.

**Table 8:** Comparison of mean simulated and observed days to flowering, days to maturity and grain yield of faba bean varieties during model validation.

Variable Name	Faba bean varieties											
	Gora						Tumsa					
	Ob	Sim	R^2^	RMSE	RMSEn	d-stat	Ob	Sim	R^2^	RMSE	RMSEn	d-stat
DF	53	54	0.58	1.58	2.95	0.795	54	54	0.649	0.76	2.92	5.39
DM	126	125	0.89	1.94	1.54	0.854	132	133	0.77	1.32	0.99	0.863
Yield (kg/ha)	2700	2692	234.4	8.68	0.974	0.88	3064	3191	0.84	280.96	8.98	0.937

Note: DF-Days to flowering, DM- Days to maturity period, Ob- Observed, Sim- Simulated.

### 4.5 Projection of faba bean yield under future climate change

**Figure 11:**
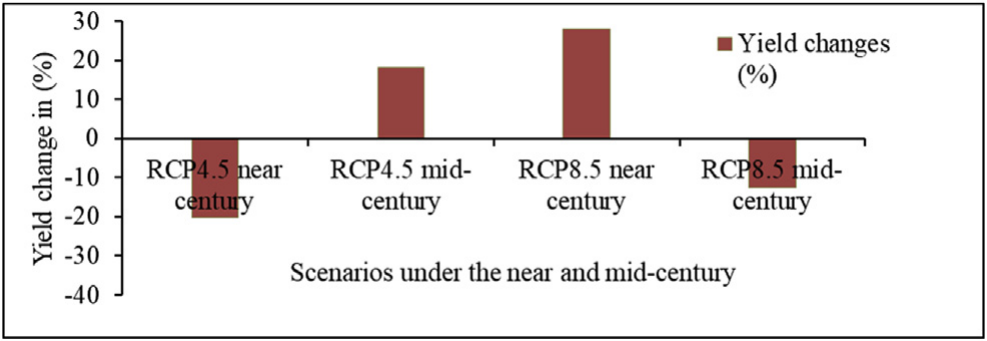
Projected yield change (%) of Gora variety from baseline mean under RCP 4.5 and RCP 8.5 by near-and mid-century over Welmera area.

**Figure 12:**
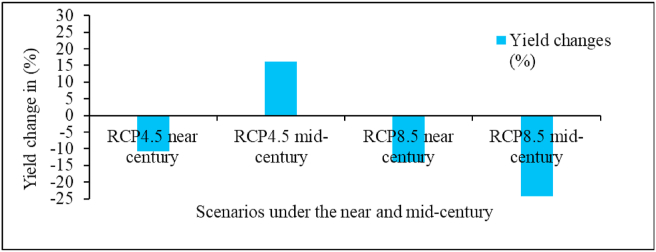
Projected yield change (%) of Tumsa variety from baseline mean under RCP4.5 and RCP8.5 by the near century and mid-century over Welmera area.

Simulation results of yield of both faba bean varieties in 2030s and 2050s under RCP 4.5 and RCP 8.5 scenarios are shown in Figures 11 and 12. The result revealed that there will be an increase and a decrease in the yield of both faba bean varieties in the end of century under medium and high emission scenarios. Gora variety will experience an increase of yield from the baseline yield (2415 kg/ha) by 18.24% and 28.03% under RCP 4.5 in mid-century and RCP 8.5 in near-century, respectively (Figure 11). However, in 2030s and 2050s, yield will decline under RCP4.5 (near-century) and Rcp 8.5 (mid-century).

Future climate projection has a positive impact on the yield of Tumsa variety by 16.18% in 2050s under RCP 4.5 scenario. However, a decline of 14.05% and 24.19% from the baseline yield of 2561 kg/ha is projected for 2030s and 2050s under RCP 8.5 scenarios, respectively (Figure 12). The decrease in yield might be due to rising temperature and associated negative impacts. Short episodes of high temperature (minimum or maximum) can essentially impede crop development and thus impact crop yield independent of any substantial changes in mean temperature (Wheeler et al., 2006). Changes in temperature regimes can interfere with reproductive stage of plant development and adversely affect crop yield (Hatfield and Prueger, 2015).

Precipitation projections for Africa are less certain than the corresponding temperature features. This is especially due to a lack of observational data and inconsistencies between different observed precipitations datasets (Niang et al., 2014). Overall, rainfall is expected to increase in the upcoming decades despite declines in certain monthls while both minimum and maximum temperature will increase in the study area in the future. The findings of the study showed that Tumsa variety will incur more yield loss under RCP 8.5 in mid-century, which could be due to a range of non linear processes that impact yield individually or interact to constrain crop production.

## 5 Conclusion and recommendation

This study was carried out at Welmera area, Central highland of Ethiopia to model the impacts of climate change on faba bean (*Vicia faba* L.) production. Projected future annual and seasonal rainfall will increase by 2030s and 2050s under RCP 4.5 and RCP 8.5 scenarios. As compared to the baseline period, annual rainfall by the 2030s will increase up to 50% under RCP 4.5 scenario. Future projected average seasonal rainfall total of *belg* will increase substantially under both RCPs scenarios. The projected highest mean monthly rainfall during faba bean growing season will be in July whereas, rainfall will decrease dramatically in August during 2030s and 2050s under both RCPs scenarios, which might adversely affect faba bean yield. Minimum and maximum temperatures are projected to increase under both scenarios and during both time slices.

Overall, CROPGRO model validation for yield of faba bean (Gora and Tumsa) varieties showed excellent performance in simulating days to flowering, days to physiological maturity and grain yield than calibration model. The d-statistic for the both varieties had good agreement between simulated and observed days to anthesis, days to maturity and yields (kg/ha).

The projected future climate will have variable impact on the two faba bean varieties. Gora variety will increase up to 28% in near-century, but faba bean yield will decline under RCP 4.5 (near-century) and RCP 8.5 (mid-century). The projected future climate change will have highly negative impact (a decline of up to 24%) on the yield of Tumsa variety under various concentration pathways, whereas an increase of 16.18% is projected to occur under RCP 4.5 (mid-ccentury) in 2050s.

This study covered only a single district and its surrounding, which could be considered as a limitation thus making it difficult to generalize. Moreover, there is limitation of data quality control due to the absence of latest instruments at gauge station for accurate measurement of weather in the area. Further studies that consider different climate and crop models that project future climate and simulate crop production are necessary to better understand the impact of future climate on faba bean production in the study area.

## Declarations

### Author contribution statement

Girma Asefa Bogale: Conceived and designed the experiments; Performed the experiments; Analyzed and interpreted the data; Wrote the paper.

Mengistu Mengesha Maja: Conceived and designed the experiments; Wrote the paper.

Gebre Hadgu Gebreyohannes: Conceived and designed the experiments; Analyzed and interpreted the data; Wrote the paper.

### Funding statement

This research did not receive any specific grant from funding agencies in the public, commercial, or not-for-profit sectors.

### Data availability statement

Data will be made available on request.

### Declaration of interests statement

The authors declare no conflict of interest.

### Additional information

No additional information is available for this paper.
